# Age-related macular degeneration and progression of coronary artery calcium: The Multi-Ethnic Study of Atherosclerosis

**DOI:** 10.1371/journal.pone.0201000

**Published:** 2018-07-18

**Authors:** Antonio B. Fernandez, Kevin D. Ballard, Tien Y. Wong, Mengye Guo, Robyn L. McClelland, Gregory Burke, Mary Frances Cotch, Barbara Klein, Matthew Allison, Ronald Klein

**Affiliations:** 1 Division of Cardiology, Heart and Vascular Institute, Hartford Hospital, Hartford, CT, United States of America; 2 Department of Medicine, University of Connecticut, Farmington, CT, United States of America; 3 Department of Kinesiology and Health, Miami University, Oxford, OH, United States of America; 4 Singapore Eye Research Institute, Singapore National Eye Center, Duke-NUS Medical School, National University of Singapore, Singapore; 5 Department of Biostatistics, University of Washington, Seattle, WA, United States of America; 6 Wake Forest University Health Sciences, Department of Public Health Sciences, Winston-Salem, NC, United States of America; 7 Division of Epidemiology and Clinical Applications, NIH Intramural Research Program, National Eye Institute, National Institutes of Health, Bethesda, MD, United States of America; 8 Department of Ophthalmology & Visual Sciences, University of Wisconsin, Madison, WI, United States of America; 9 University of California San Diego, La Jolla, CA, United States of America; Universita degli Studi di Firenze, ITALY

## Abstract

**Background:**

Age-related macular degeneration (AMD) shares many similarities with cardiovascular disease (CVD) pathophysiology. We sought to determine the relationship of AMD to the progression of coronary artery calcium (CAC) using data from the Multi-Ethnic Study of Atherosclerosis (MESA).

**Methods:**

Our cohort consisted of 5803 adults aged 45 to 84 years free of known cardiovascular disease (CVD). Retinal photographs were taken during visit 2 (Aug 2002-Jan 2004). CAC was measured with computed tomography at visit 1 (July 2000-Aug 2002) and visit 5 (April 2010-Dec 2011) and changes between visits were determined.

**Results:**

Participants were categorized as with (n = 244) and without AMD (n = 5559) at visit 2. At visit 5, 92 participants with and 2684 without AMD had CAC scores. Among those with detectable CAC at baseline (>0 at visit 1), CAC progression was greater in persons with compared to those without AMD after multivariable adjustment (530 ± 537 vs. 339 ± 426 Agatston units, P<0.01).

**Conclusions:**

The presence of AMD in a diverse population without known clinical CVD independently predicted higher 10-year CAC progression in participants with baseline CAC >0. The retinal exam might be a useful tool for pre-clinical assessment and prevention of CVD events.

## Introduction

Age-related macular degeneration (AMD) is a leading cause of vision loss in the US [1]. It is estimated it will affect 288 million adults by 2040 [2]. The presence of AMD is associated with various traditional cardiovascular disease (CVD) risk factors, including age [3], hypercholesterolemia [4], hypertension [5], and cigarette smoking [6], suggesting that the pathophysiology of AMD and atherosclerotic CVD may be similar [7]. Additionally, AMD has been reported to be associated with increased risk of stroke [8,9],coronary heart disease (CHD) [10] and all-cause mortality [11]. Data from population-based studies evaluating AMD as a risk indicator for cardiovascular events independent of traditional CVD risk factors have been inconsistent [12,13,14,15,16]. Non-invasive cardiovascular testing and imaging techniques, such as coronary artery calcium (CAC), are indicators of subclinical atherosclerosis. These indicators have been associated with higher risk for future CVD events and have been suggested as surrogate measures of CVD [17,18]. Thus, the objective of this study was to determine the association of AMD with the progression of CAC as a subclinical CVD marker and whether any observed relationship was independent of traditional CVD risk factors in a healthy, diverse population participating in the Multi-Ethnic Study of Atherosclerosis (MESA). We hypothesized that the presence of AMD would predict the progression of CAC.

## Materials and methods

### Participants and study design

The MESA is a prospective longitudinal study with the purpose of identifying risk factors for subclinical CVD and its progression to clinical events [19]. The MESA cohort consists of a diverse population sample of 6814 men and women aged 45 to 84 years at baseline (Exam 1; July 2000 to July 2002) who were recruited from 6 field centers: Baltimore, MD; Chicago, IL; Forsyth County, NC; Los Angeles, CA; New York, NY; and St. Paul, MN. Individuals with a known history of CVD at baseline were excluded. Known history of CVD included myocardial infarction, angina, heart failure, stroke, or transient ischemic attack or the following procedures: coronary artery bypass graft, angioplasty, or other vascular surgeries. The cohort for the present investigation consisted of adults free of known CVD who underwent retinal photography during the first follow-up examination (Exam 2) conducted from August 2002 to January 2004. The tenets of the Declaration of Helsinki were adhered to, and each participating field center was granted all necessary institutional review board approvals. Written informed consent was obtained from each participant. This study was registered at clinicaltrials.gov as NCT00005487.

### Retinal examination

Retinal photography was performed using a standardized protocol [20]. Both eyes of each participant were photographed using a 45°, 6.3-megapixel digital non-mydriatic camera (Canon, Lake Success, NY). Two photographic fields were taken of each eye, the first centered on the optic disc and the second centered on the fovea. The macula area was graded at the University of Wisconsin Ocular Epidemiology Research Center for AMD features using a standard AMD grading protocol [20]. Features of AMD included drusen size, type, and area; increased retinal pigment; retinal pigment epithelial depigmentation; pure geographic atrophy; and signs of exudative macular degeneration (subretinal hemorrhage, subretinal fibrous scar, retinal pigment epithelial detachment, and/or serous detachment of the sensory retina or laser or photodynamic treatment of for neovascular AMD). Graders were masked with respect to information about the participant and each retinal image was graded twice (preliminary and detail grading) using a modification of the Wisconsin Age-Related Maculopathy Grading scheme [21].

Early AMD was defined by: 1) either the presence of any soft drusen (distinct or indistinct) and pigmentary abnormalities (either increased retinal pigment or retinal pigment epithelium depigmentation); or 2) the presence of a large soft drusen ≥125 μm in diameter with a large drusen area (>500 μm in diameter circle); or 3) large (≥125 μm in diameter) soft indistinct drusen. Late AMD was defined by the presence of geographic atrophy or retinal pigment epithelial detachment, subretinal hemorrhage or visible subretinal new vessels, or subretinal fibrous scar or laser treatment scar for AMD. Any AMD was defined by the presence of early or late AMD [22].

### Coronary artery calcium

Acquisition, interpretation and measurement variance of CAC scans have been reported previously [23,24]. In the present study, our sample for incident CAC and CAC progression only included participants who had CAC measured at both baseline and Exam 5 (April 2010-December 2011). Incident CAC was defined as a baseline calcium score of 0 (zero) and available repeat CAC score > 0. Progression of CAC was determined for those participants with baseline CAC scores greater than 0 (zero) and available repeat CAC whose score was higher than the participant’s baseline value. Any increments from the baseline CAC score were considered progression of CAC.

### Statistical analyses

Data are means ± SD or proportions for continuous and categorical variables, respectively, with the exception of baseline CAC score which is presented as median [IQR]. After age adjustment, means were compared via linear regression, categorical variables via logistic regression (with AMD as the endpoint), and medians using quantile regression. Demographic variables, CVD risk factors, and CAC at baseline were compared between AMD groups using one-way analysis of variance (ANOVA) or χ^2^ tests. Among the participants with positive CAC at baseline, robust linear regression coefficients and corresponding 95% confidence intervals were estimated to compare the progression of CAC between the group of participants with AMD and without AMD, adjusting for age, gender, race, level of education, systolic blood pressure, hypertension medication, lipid lowering medication, smoking status, diabetic status, serum total cholesterol, HDL cholesterol, and C-reactive protein.

## Results

**Fig 1:**
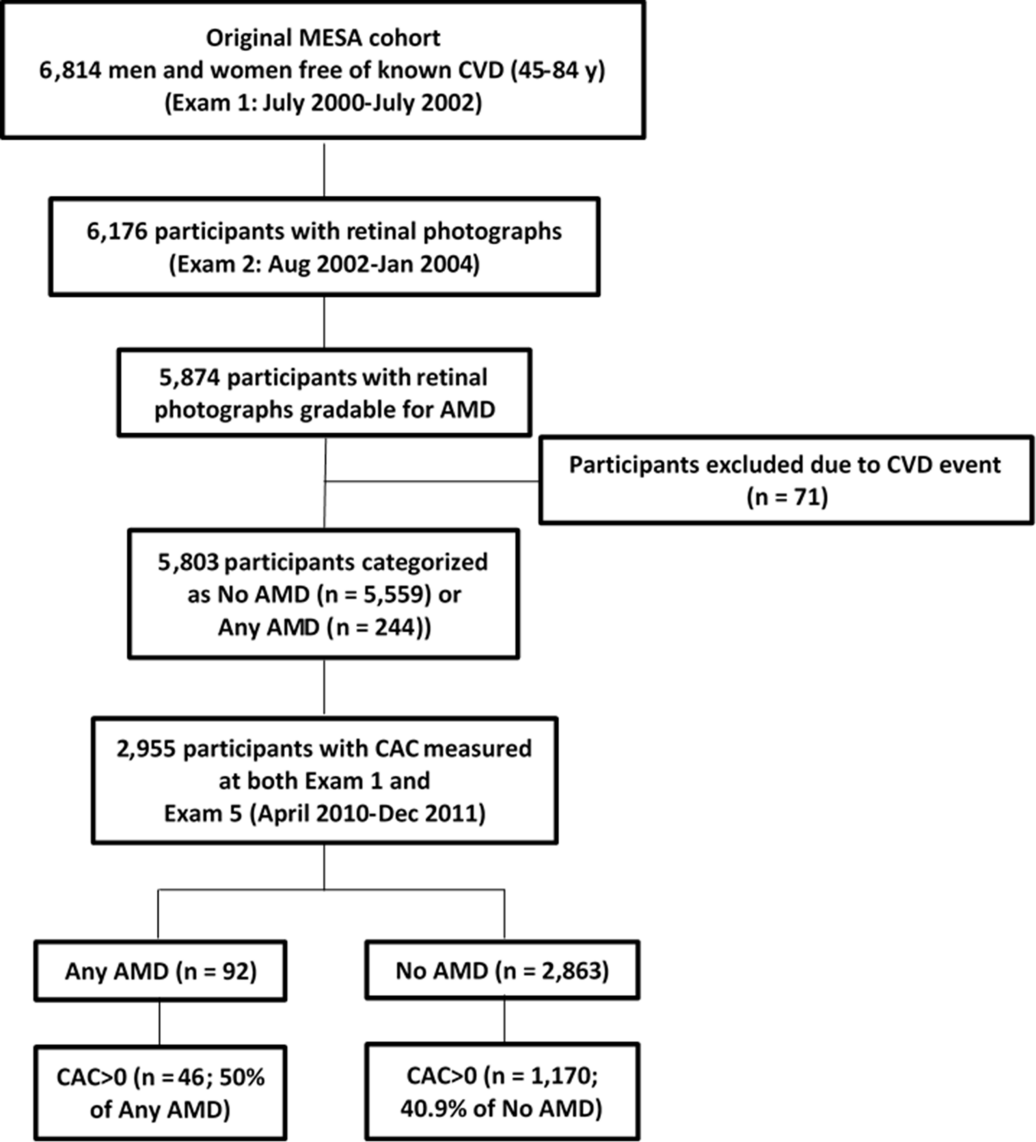
Flow diagram describing the studied cohort.

Of the 6176 participants who had retinal photographs acquired, 5874 (95%) had images gradable for AMD (**Fig 1**). Excluding those with CVD events prior to the first follow-up visit, our cohort consisted of 5803 participants at baseline (**S1 Table**). Of these, 219 persons (3.8%) had early AMD and 25 (0.4%) had late AMD. Thus, the presence of any AMD was detected at baseline in 244 (4.2%) participants, while 5559 persons were categorized as no AMD (**S1 Table**).

**Fig 2:**
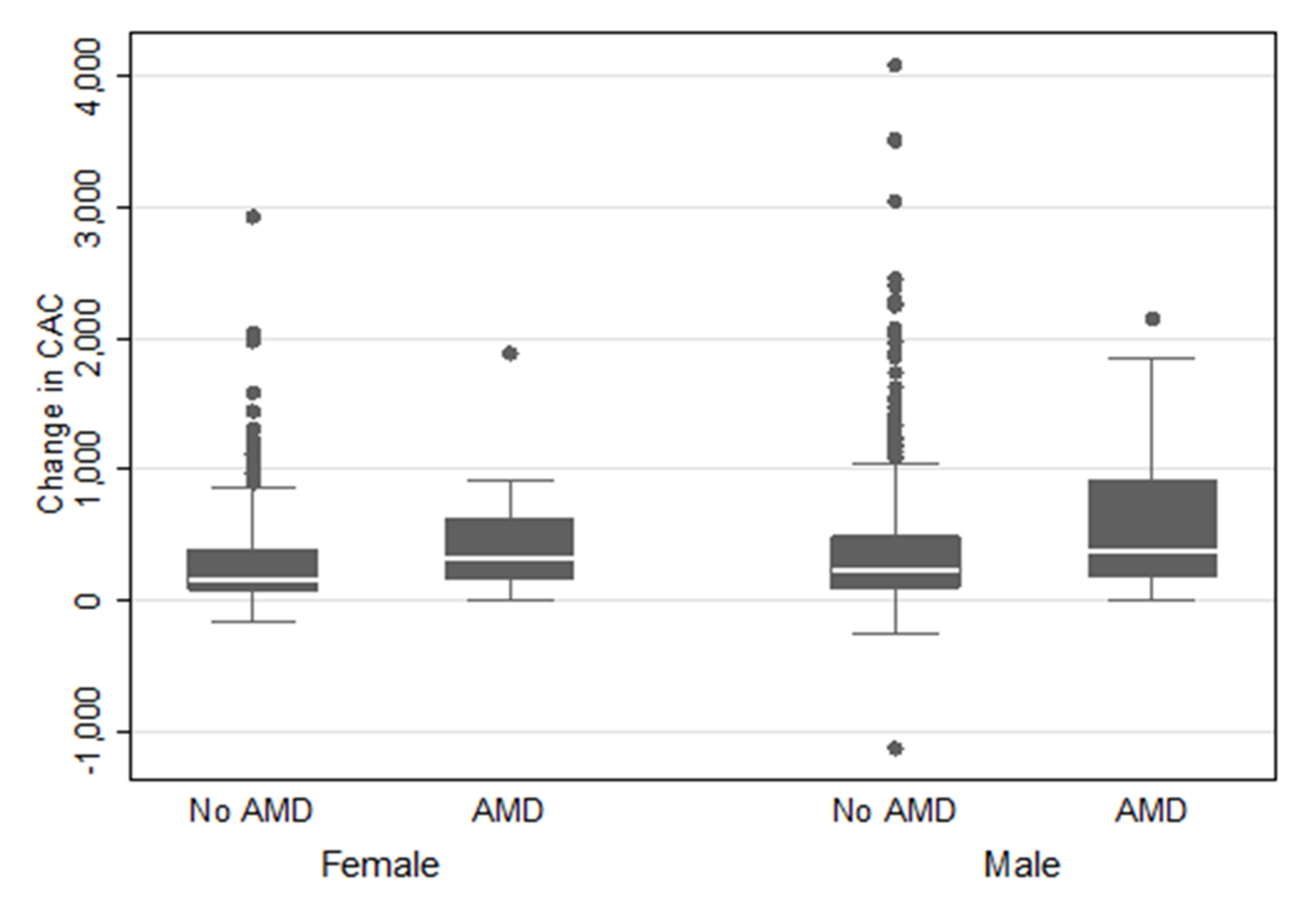
Change in CAC score between Exam 2 and Exam 5 by AMD status and sex. CAC = coronary artery calcium; AMD = age-related macular degeneration.

Of the 2955 participants who had CAC measured at both baseline and Exam 5, 92 and 2863 were categorized as with and without AMD, respectively (**Table 1**). CAC scores were significantly greater in participants who had AMD at baseline (median 143.1 [IQR 20.6, 384.2] versus 57.7 [15.2, 196.0] Agatston units; age-adjusted P-value = 0.021). There were 1170 (40.9%) and 46 (50%) participants with detectable CAC (CAC>0) at baseline in the group without AMD and with AMD (P>0.05), respectively (**S1 Table**). There was no difference in the 10-year incidence of the development of CAC as a function of AMD status (**Table 2**). After multivariable adjustment, and in participants with baseline CAC>0, 10-year CAC progression was greater in the participants with AMD compared to participants without AMD (P<0.01) (**Table 2**). In participants with baseline CAC>0, 10-year CAC progression was greater in participants with early AMD (n = 39) compared to participants without AMD (n = 1170) (P = 0.018) (**Table 3**). Differences in the progression of CAC between types of AMD (early vs. late) could not be ascertained due to limited power. Men and women had a similar association between AMD and CAC progression (**Fig 2**).

**Table 1:** Baseline characteristics from participants with data on AMD who had CAC measured at both baseline (Exam 1) and Exam 5.

Characteristics	Overall Cohort (n = 2955)^#^	No AMD (n = 2863)^#^	Any AMD (n = 92)^#^	Total n
Age*, years	59.6 (9.2)	59.3 (9.1)	66.4 (9.4)	2955
Male, n (%)	1379 (46.7)	1332 (46.5)	47 (51.1)	2955
Race*				
Caucasian, n (%)	1148 (38.8)	1103 (38.5)	45 (48.9)	2955
Chinese, n (%)	349 (11.8)	340 (11.9)	9 (9.8)	
African Americans, n (%)	801 (27.1)	787 (27.5)	14 (15.2)	
Hispanics, n (%)	657 (22.2)	633 (22.1)	24 (26.1)	
High school graduate, n (%)	2552 (86.5)	2480 (86.7)	72 (79.1)	2950
Total cholesterol mg/dL	194.2 (34.9)	194.3 (35.0)	192.1 (32.8)	2947
HDL cholesterol mg/dL	51.0 (14.7)	50.9 (14.7)	53.4 (16.4)	2944
Hypertension medication, n (%)	997 (33.8)	964 (33.7)	33 (35.9)	2954
Lipid lowering medication, n (%)	448 (15.2)	433 (15.1)	15 (16.3)	2954
Diabetics, n (%)	271 (9.2)	262 (9.2)	9 (9.8)	2948
Former smokers, n (%)	1057 (35.8)	1021 (35.7)	36 (39.6)	2950
Current smokers, n (%)	355 (12.0)	345 (12.1)	10 (11.0)	2950
CAC = 0, n (%)	1739 (58.8)	1693 (59.1)	46 (50.0)	2955
1–99, n (%)	750 (25.4)	730 (25.5)	20 (21.7)	
100–399, n (%)	306 (10.4)	290 (10.1)	16 (17.4)	
≥400, n (%)	160 (5.4)	150 (5.2)	10 (10.9)	
CAC score among those CAC>0*, median (IQR)	58.6 (15.4, 200.7)	57.7 (15.2, 196.0)	143.1 (20.6, 384.2)	1216

Data are means (SD) or proportions unless otherwise indicated. Abbreviations: AMD = age-related macular degeneration; CAC = coronary artery calcium; IQR = interquartile range.

^#^Sample size when there is no missing data.

All tests controlled for age, with

* indicating significance at the p<0.05 level.

**Table 2:** Ten-year CAC progression by baseline AMD status (No AMD vs. Any AMD).

Baseline CAC = 0	Incident CAC, n (%)	Age, gender, time between CT scans adjusted OR (95% CI)	Multivariable* adjusted OR (95% CI)
No AMD(n = 1693)	761 (45%)	ref	ref
Any AMD(n = 46)	21 (46%)	0.71 (0.38, 1.31)	0.78 (0.42,1.47)
**Baseline CAC>0**	**Progression of CAC (CAC5-CAC1) (mean ± SD)**	**Robust regression model, Age, gender, time between CT scans adjusted (diff (se), p)**	**Multivariable robust regression model,(diff (se), p)**
No AMD(n = 1170)	339 ± 426	ref	ref
Any AMD(n = 46)	530 ± 537	101 (40), 0.011	114 (39), 0.004

Abbreviations: AMD = age-related macular degeneration; CAC = coronary artery calcium.

*adjusting for time between scans, age, gender, race, SBP, hypertension med, smoking status (never, former vs. current), total cholesterol, HDL cholesterol, lipid lowering med, CRP, diabetic status and education (high school or higher).

**Table 3:** Ten-year CAC progression by baseline AMD status (No AMD vs. Early/Late AMD).

Baseline CAC = 0	Incident CAC, n (%)	Age, gender, time between CT scans adjusted OR (95% CI)	Multivariable* adjusted OR (95% CI)
No AMD (n = 1693)	761 (45%)	ref	ref
AMD early (n = 44)	20 (45%)	0.70 (0.38, 1.31)	0.78 (0.41, 1.48)
AMD late (n = 2)	1 (50%)	0.69 (0.04, 11.60)	0.84 (0.05, 15.01)
**Baseline CAC>0**	**Progression of CAC** **(CAC5-CAC1)** **(mean ± SD)**	**Robust regression model, Age, gender, time between CT scans adjusted(diff (SE), p-value)**	**Multivariable robust regression model,(diff (SE), p-value)**
No AMD (n = 1170)	339 ± 426	ref	ref
AMD early (n = 39)	529 ± 532	102 (43), 0.018	116 (42), 0.006
AMD late (n = 7)	539 ± 607	99 (99), 0.319	104 (97), 0.283

Abbreviations: AMD = age-related macular degeneration; CAC = coronary artery calcium.

*adjusting for time between scans, age, gender, race, SBP, hypertension med, smoking status (never, former vs. current), total cholesterol, HDL cholesterol, lipid lowering med, CRP, diabetic status.

## Discussion

The present study demonstrates that the presence of AMD is associated with a greater 10-year CAC progression after multi-variable adjustment in a diverse population. Our data suggest that participants with AMD free of known clinical CVD with a CAC = 0 had no increase in the incidence of CAC. Participants with a baseline CAC score>0 had greater CAC progression as compared to those without AMD after a mean follow-up of 10.4 years in our cohort. This subclinical progression was independent of traditional CVD risk factors. This finding is supported by some [13,14,16,25,26], but not all [9,15,27], studies evaluating AMD and CVD/CHD events.

There are few published studies evaluating the contribution of baseline AMD to the development of clinical atherosclerotic CVD [13, 14,16,25,26,9,15,27]. An evaluation from the Atherosclerosis Risk in Communities (ARIC) study dataset demonstrated that participants early macular degeneration had a higher incident stroke hazard ratio (HR = 1.85, 95% CI, 1.19–2.87) than individuals without AMD [9]. Individuals with late AMD were three times more likely to have an incident coronary artery disease (CAD) event (RR = 3.05, 95% CI, 1.14–8.17 with a 10-year cumulative incidence, 30.9%) as compared to participants without late AMD (10-year cumulative incidence, 10%) [16].This supports the notion that the more severe forms of AMD might have an association with CAD events. In the Blue Mountain Eye Study participants younger than 75 years with baseline early AMD had a 2.26-fold increased risk of CAD mortality when compared to age and sex matched controls [15]. Adjustments of relative risk by serum lipid levels and hypertension treatment were not reported.

Our group found no increased risk of clinical CVD events in patients with baseline AMD in a previous investigation also using the MESA dataset [14]. It is possible that the MESA cohort design could explain the discrepancy found with the results from other cohorts. The MESA participants had no clinical CVD and were likely healthier than those in other studies. Therefore, our current hypothesis sought to determine whether differential patterns of subclinical CVD, as measured by CAC scores changes, exist in our cohort. To our knowledge, this is the first study correlating baseline AMD and CAC progression.

CAC scores have been evaluated for their contribution to CVD risk prediction. The 2013 American College of Cardiology/American Heart Association cholesterol guidelines introduced the use of the new risk prediction Pool Cohort Equation and CAC to improve atherosclerotic CVD risk prediction and risk-based treatment strategies in primary CVD prevention [28]. Data from the MESA showed that CAC independently predicted CVD events and improves risk discrimination over and beyond the Pool Cohort Equation and the traditional Framingham risk equation enhancing CVD risk prediction [29,30].

Progression of vascular calcification has the potential to better capture the temporal exposure to risk factors compared with a single baseline score. It has been suggested that a baseline CAC can be thought of as a single point on an atherosclerosis-versus-time curve, whereas progression correlated with the slope of that curve [31]. Baseline scores might reflect atherosclerotic plaque burden, whereas progression might provide insight into ongoing current disease activity. Rapid progression of CAC is independently predictive of mortality [32].

3-hydroxy-3-methylglutaryl coenzymeA (HMG-CoA) reductase inhibitors or “statins” can stabilized coronary artery plaques by increasing its calcium composition and this relationship could have an inverse relationship with CVD events [17]. This paradoxical finding is presumed to be a healing phenomenon that occurs when lipid is removed from the arterial wall, resulting in more calcium deposition. It is possible that in our study, the increase in CAC progression in the AMD group was due to increased statin use. In our cohort, the prevalence of lipid-lowering medication use was higher in participants with (16.4%) versus without AMD (15.7%). Nonetheless, some of the participants not on lipid-lowering agents at baseline were prescribed these medications during follow-up. The two-time point design of our CAC progression model did not allow incorporating new statin use as a time-varying covariate. Thus, it is possible that our findings could reflect a closer medical care (including greater statin use) of the AMD group and statin-induced CAC progression denoting more calcified but stable plaque burden.

Another limitation of our study includes the failure to capture a larger proportion of individuals with AMD. The MESA participants had no clinical CVD and were likely healthier than those in other studies. This could explain the lower proportion of AMD participants in our cohort and the discrepancy found with our previous investigation of clinical outcomes and the results from other cohorts. Lastly, the AMD assessment was made at visit 2 of the MESA (August 2001-January 2004) but the baseline CAC was measured at visit 1 (July 2000-August 2002). This difference in the timing of baseline measures may have impacted our incidence analysis as it is possible that CAC may have developed between visits 1 and 2. In conclusion, the association of AMD with 10-year CAC progression in the MESA suggests a greater progression of CVD. Our study adds to the previous conflicting reports regarding the clinical importance of AMD in CVD prediction [12,13,14,15,16], and supports the notion that AMD may be a local manifestation of systemic processes and not confined to the deeper layers of the retina [33]. The higher progression of CAC in the AMD group suggests that AMD might be a player in the progression subclinical CVD which could be important for clinicians who employ serial screening techniques to assess changes in risk or progressive disease states. A retinal exam might be a useful tool for pre-clinical assessment of microvascular processes that underlie the development of CVD. In patients already diagnosed with AMD, communication between the internist, an ophthalmologist and potentially a cardiologist might be beneficial when managing the care of each condition. However, our finding has not correlated with clinical outcomes in our cohort yet. The clinical evidence linking AMD and CAD remains inconclusive and firm recommendations should await the results of dedicated clinical outcomes studies in patients with AMD.

## Supporting information

S1 Table Baseline characteristics for all MESA participants with data on AMD. (DOCX)

## Abbreviations

AMD: age-related macular degeneration
ANOVA: one-way analysis of variance
CAC: coronary artery calcium
CHD: coronary heart disease
CVD: cardiovascular disease
MESA: the Multi-Ethnic Study of Atherosclerosis
