# Supplementary material for: Age-related macular degeneration and progression of coronary artery calcium: The Multi-Ethnic Study of Atherosclerosis
Source: PLoS One. 2018 Jul 18;13(7):e0201000. doi: 10.1371/journal.pone.0201000 (PMC6051657; doi:10.1371/journal.pone.0201000)
Supplement: S1 Table — (DOCX) [file pone.0201000.s001.docx]

**S1 Table. Baseline characteristics for all MESA participants with data on AMD.**

| **Characteristics** | **Overall Cohort**  **(n = 5808)^#^** | **No AMD**  **(n = 5564)^#^** | **Any AMD**  **(n = 244)^#^** | **Total n** |
| --- | --- | --- | --- | --- |
| Age, years* | 61.4 (10.0) | 61.1 (9.9) | 69.5 (9.9) | 5808 |
| Male, n (%) | 2760 (47.5) | 2640 (47.4) | 120 (49.2) | 5808 |
| Race* |  |  |  | 5808 |
| Caucasian, n (%) | 2287 (39.4) | 2166 (38.9) | 121 (49.6) |  |
| Chinese, n (%) | 690 (11.9) | 658 (11.8) | 32 (13.1) |  |
| African Americans, n (%) | 1567 (27) | 1530 (27.5) | 37 (15.2) |  |
| Hispanics, n (%) | 1264 (21.8) | 1210 (21.7) | 54 (22.1) |  |
| High school graduate, n (%) | 4827 (83.4) | 4635 (83.6) | 192 (79.7) | 5786 |
| Total cholesterol mg/dL | 194.2 (35.5) | 194.2 (35.5) | 192.6 (33.6) | 5791 |
| HDL cholesterol mg/dL | 51 (14.8) | 50.9 (14.8) | 52.9 (15.1) | 5788 |
| Hypertension medication, n (%) | 2067 (35.6) | 1966 (35.4) | 101 (41.4) | 5805 |
| Lipid lowering medication**, n (%) | 914 (15.7) | 874 (15.7) | 40 (16.4) | 5805 |
| Diabetics**, n (%) | 650 (11.2) | 631 (11.4) | 19 (7.8) | 5790 |
| Former smokers, n (%) | 2107 (36.4) | 2002 (36.1) | 105 (43.4) | 5792 |
| Current smokers, n (%) | 740 (12.8) | 720 (13) | 20 (8.3) | 5792 |
| CAC = 0, n (%) | 3053 (52.6) | 2959 (53.2) | 94 (38.5) | 5808 |
| 1-99, n (%) | 1510 (26) | 1449 (26) | 61 (25) |  |
| 100-399, n (%) | 737 (12.7) | 683 (12.3) | 54 (22.1) |  |
| ≥400, n (%) | 508 (8.7) | 473 (8.5) | 35 (14.3) |  |
| CAC score among those CAC>0, median (IQR) | 80.1 (20.3, 279.7) | 76.6 (19.9, 272.4) | 151.1 (36.7, 384.2) | 2755 |
| Mean of left and right mean cIMT, mm | 0.8 (0.2) | 0.7 (0.2) | 0.8 (0.3) | 2822 |
| FMD, % | 4.4 (2.9) | 4.4 (2.9) | 3.7 (2.6) | 3200 |
| CRP, mg/L | 3.6 (5.4) | 3.6 (5.3) | 3.7 (6.2) | 5768 |
| IL-6, pg/mL | 1.5 (1.2) | 1.5 (1.2) | 1.7 (1.3) | 5652 |
| Fibrinogen, mg/dL | 343.8 (72) | 343.5 (72) | 350.4 (70.8) | 5772 |
| TNF-α, pg/mL | 1335.6 (410.8) | 1330.1 (403.9) | 1452.7 (524) | 2461 |
| Subset for cIMT  Baseline cIMT | 0.750 (0.178) | 0.747 (0.174) | 0.832 (0.257) | 2776 |
| Subset for ABI  Baseline ABI<0.9 (PAD) | 91/4114 (2%) | 83/3976 (2%) | 8/138 (6%) | 4114 |

Data are means (SD) or proportions unless otherwise indicated. Abbreviations: AMD = age-related macular degeneration; CAC = coronary artery calcium; CCA = common carotid artery; cIMT = carotid artery intima-media thickness; CRP = C-reactive protein; FMD = flow mediated dilation; IL-6 = interleukin-6; IQR = interquartile range; TNF- α = tumor necrosis factor- α.

^#^Sample size when there is no missing data. *P<0.05 by the age-adjusted test; raw test for age, gender and race. **After adjusting for age, participants with any AMD had significantly higher prevalence of lipid-lowering medication use and fewer diabetics than participants with no AMD.
